# Notes and News

**Published:** 1889-04-13

**Authors:** 


### Notes and News.

Collected and Communicated.

The British Association for the Advancement of Science will hold its annual meeting at Newcastle-on-Tyne this year, where the members will be sure of a hearty welcome and some interesting excursions. Prof. W. H. Flower has been elected president, and the section of biology will be presided over by Dr. Burdon-Sanderson. The meetings will be held between September 11 and 19, and several papers have been already promised. Mr. A. T. Atchinson's (the secretary's) resignation from ill health leaves a vacancy worth £300 per annum.

The story of the dog who has applied for admission at Bristol Royal Infirmary is doubtless meant to be pathetic, but is simply laughable. The hero who took his wounded companion to King's College Hospital was an authentic dog, but his story has given rise to more or less discreditable imitators. The *St. James's Gazette* made a clever remark when it asked how the dog knew the hospital was a place for healing. The King's College dog had been attended there before, and simply made his way back to where he had formerly found relief, and we think his intelligence was more worthy of remark than the mere accident which caused a wounded dog at Bristol to sneak into the Infirmary grounds.

The annual report of St. Mary's Hospital tells a tale of satisfactory progress. Last year the in-patients numbered 3,293, as compared with 3,315 in 1887; out-patients, 14,487 against 14,608; casualties, 12,047 against 11,600; and maternity cases 545, as compared with 429 the previous year. The number of beds available remained unaltered—namely, 281. The average number of beds occupied daily was higher than in the previous year, being 250·5 against 242·8. The greatest number in the hospital on any one day was 286, and the smallest 171, and the mean duration of the stay of in-patients was 27·85 days against 26·74 in 1887. The thanks of the governors are specially given to many friends who have contributed largely to the funds, and this list is headed by the name of Lord Randolph Churchill, who so kindly presided at the festival dinner last May. The hospital has made another great improvement during the year in enlarging its sphere of work by the constitution of an out-patient children's department. The need of such a department has long been felt, and its establishment is an important addition to the hospital, both as a charity for the relief of the sick poor and as a medical school. Mr. Edmund Owen, one of the surgeons of the hospital, and Dr. Sydney Phillips, one of the out-patient physicians, have been appointed surgeon and physician respectively. The medical school also shows great improvement and progress. The nursing staff under Miss Medill continues to work well, and excellent training is given to probationers.

Amongst the Saturday afternoon lectures at South Kensington Museum have been two by Prof. Muybridge, of Pennsylvania, on "Animal Locomotion." The lectures were illustrated by instantaneous photographs of animals in motion, and anything stranger than such a photograph of a trotting horse or a flying bird it is impossible to imagine. For grace, however, we still prefer the artistic representations of animals in motion, which, if incorrect, at least do not look impossible.

Abingdon is one of our best managed cottage hospitals. It was built by Mr. J. C. Clarke, who still acts as its president, and is supported by the local benefit societies, churches, and the ladies, who make large collections on its behalf. During last year 98 patients were treated in the hospital and six deaths occurred. The treasurers have a balance in hand of £72, and £2,000 has been invested by the Charity Commissioners. The medical men attend the hospital willingly, and the matron, Miss Miller, renders services which are duly appreciated. It is to be hoped that the hospital may long pursue the present even tenor of its way.

There is great excitement at Harrogate about the promised visit of Prince Albert Victor. The new pile of buildings which is rising on the site of the old Bath Hospital will be finished in July, and Prince Albert Victor, who seems specially fond of Yorkshire, has promised to open it, and also to open the Rawson Convalescent Home. A sub-committee of the hospital has been appointed to arrange a public demonstration on an elaborate scale, to celebrate the visit of His Royal Highness. The two institutions which Prince Albert Victor will open are a credit to one of our best inland health resorts, and we hope the people of Harrogate will give their Royal guest a hearty welcome.

One of the staff of the *Liverpool Porcupine* is doing the rounds of the local hospitals, and giving a description of one of these institutions every week. In dealing with the Stanley Hospital he calls attention to the fancy fair to be held in its aid at Whitsuntide, when it is hoped to raise £4,000 to clear off the present debt. Dr. Costine and Dr. Sheldon are working hard towards this end. But this roving reporter does not confine himself to beaten paths; he makes his way into an operating theatre while the surgeons are busy, and describes the work done there from a stranger's point of view. There can be no doubt that these articles in local papers arouse interest, but the lesson they teach is rather that the hospital which is most easily inspected, most freely open to visitors, is best supported. Better than all reports, and appeals, and newspaper paragraphs is to give a hearty welcome to those who wish to see round the wards, for the pale faces of the patients will plead better than any words.

One of the greatest wants for the sick in England just now is some form of institution between a hospital and a convalescent home, where cases which fit none of the existing institutions could be received. We are glad to note that the needs of the children in this direction have been recognised, and that a convalescent hospital has been opened at Bushey Heath, where such cases as caries of the spine, diseases of the joints, etc., are taken. The hospital is under the charge of two of the late sisters of Charing Cross, and is a pleasant little cottage with a cheerful look out. A minimum charge of 7s. per week is made for each child, and none can be received for a period of less than three weeks. Restrictions must come in somewhere, so infants under two and boys over ten are not admitted, but otherwise the home takes just those saddest cases which most sorely need the benefits it can bestow.

The following vacancies are announced this week, the amount of the salary in each case being given after the name of the institution:

**Resident Medical Officers.**

Brighton, Hove, and Preston Dispensary. Two House Surgeons.—£140 per annum, with apartments.
Birmingham General Dispensary. Resident Surgeon.—£150 per annum (with allowance of £30 for cabs), and furnished apartments.
Crichton Royal Institution, Dumfries. Junior Medical Assistant. Must be good at outdoor sports. — £100 per annum.
City of London Hospital for Diseases of the Chest, Victoria Park, E. House Physician.
Derbyshire Infirmary, Denbigh. House Surgeon, who must be conversant with the Welsh language. — £85 per annum, with prospect of increase; board, &c.
East London Hospital for Children, Shadwell, E. Resident Clinical Assistant. Board; no salary.
Hospital for Consumption and Diseases of the Chest, Brompton. Assistant Resident Medical Officer.—£50 per annum, and board.
Kent and Canterbury Hospital. Assistant House Surgeon and Dispenser.—£50 per annum, with board, &c.
Metropolitan Hospital, Kingsland-road, E. Senior House Surgeon.—£80 per annum, with board, &c.
Metropolitan Hospital, Kingsland-road, E. Junior House Surgeon.—£40 per annum, with board, &c.
Royal Alexandra Hospital for Sick Children, Brighton. House Surgeon.—£80 per annum, with board, &c.
Staffordshire County Lunatic Asylum, Stafford. Junior Assistant Medical Officer.—£100 per annum, with board, &c.

**Non-Resident Medical Officers.**

Birmingham Medical Aid Society. Two vacancies, each worth about £50 per annum.
Bristol General Hospital. Surgeon.
County of London. Medical Officer of Health.—£1,000 per annum.
Great Yarmouth. Medical Officer.—£80 per annum, with extra fees for surgical operations.
Portsmouth Dockyard Medical Benefit Society. Two Medical Officers for North Sandport and Southsea respectively. North Sandport appointment, £350; Southsea £200 per annum.
Sussex County Hospital. Assistant Physician.
York Friendly Societies Medical Association.—£150 per annum, with fees and allowance for cabs.

The annual meeting of the Salisbury Diocesan Institution for Trained Nurses was held on the last Tuesday in March. Of the 186 cases nursed by the association, 13, representing 35 weeks' work, were entirely gratuitous, and eight cases requiring long attendance and occupying 57 weeks had been taken at reduced rates of from 4s. to 15s. a week. Mr. B. Lancaster has bequeathed £100 to the institution, on condition that £70 should be spent on beautifying the home.

---

The compiler of the report of the Cottage Home for Little Children at Kingstown is of a poetical turn of mind. On the outer cover are two quotations, and others are scattered throughout its manifold pages. It is rather a good plan to make a hospital report interesting, and take some trouble to have it brightly written. The figures can be relegated to the last pages for the satisfaction of such people as secretaries and editors, and the opening address can appeal to more sentimental minds. Still, quotations, unless eminently suitable, are best let alone; there is no more subtle betrayer of ignorance than an inapt quotation. To return to the report of the Kingstown Home. Total admissions since the opening of the home in December, 1887, 243, not including day children; 105 applications for admission were received during last year; 31 children were admitted; nine were received into other homes; nine were otherwise provided for; and of the remainder, the applicants were over age, or otherwise unsuitable. The average number of children in the home has been 38; the annual cost of maintenance of each child is about £6.

Here is another report going in for poetry. The committee of the Hastings Hospital thus close their report: "There is, perhaps, no more cheering and delightful retrospect than the conviction of having been useful in mitigating the sufferings of others, and of giving new life and energy to those who but for timely help would have become hopelessly incurable, and have sunk into a premature grave. Many shrink from coming into contact with disease, nor is it necessary that all should do so; but it behoves those who have not hitherto given time or money for the alleviation of suffering to consider if they cannot, by pecuniary aid or otherwise, assist in arresting disease.

"O happiest work below,
Earnest of joy above,
To sweeten many a cup of woe
By deeds of holy love!"

---

As a proof of what economy can do, we would like to call attention to the following table, published by the Chesterfield and North Derbyshire Hospital:

| Average Total Cost. | £ | s. | d. | | Cost per Day for Diet. d. |
|---|---|---|---|---|---|
| In the year 1878 | 8 | 2 | 10 | .. | 11 |
| 1879 | 8 | 13 | 0 | .. | 10¾ |
| 1880 | 7 | 3 | 0 | .. | 10¼ |
| 1881 | 6 | 12 | 6 | .. | 10½ nearly |
| 1882 | 6 | 13 | 4 | .. | 10 |
| 1883 | 6 | 2 | 10 | .. | 9½ |
| 1884 | 6 | 5 | 10 | .. | 9¼ nearly |
| 1885 | 8 | 4 | 9 | .. | 9½ nearly |
| 1886 | 5 | 7 | 0 | .. | 8 nearly |
| 1887 | 5 | 9 | 5 | .. | 7½ nearly |
| 1888 | 4 | 18 | 0 | .. | 6¼ nearly |

Here we see, year by year, the expenditure decreasing, until last year the cost of each person's food (including stimulant) in the hospital was reduced to little over 6d. a day. Seeing that this means food supplied to invalids, and the officers and servants of the institution, the matron, Miss Sharkey, is very naturally commended by the committee for her good management. At this rate they will soon be fed at no cost whatever. The total number of in-patients treated last year was 269.

---

The Rev. Edward White has lately been lecturing at one of our medical schools on the spiritual effects of suffering, and seems to have reached the conclusion that painless surgery is a sign of degenerate days. Mr. White argues that the statesman would not rush so rashly into war if he knew no anæsthetics would be given to the wounded; and he also argues that a woman spared the pangs of childbirth has not such strong affection for her offspring. Surely this is rubbish? If pain is from God, is not opium also from God? If in these latter days we have lessened a little of the physical suffering of this world, it is only that we may be better able to bear the mental anguish which is ever increasing as the fierce race for wealth grows faster and more furious. Well might the savage bear a stab or a blow to the flesh with equanimity when his spirit was spared the worries and weariness of modern civilisation. Overwrought brains and overstrained nerves shrink even from physical ill, and hence the attention now given to abating mere bodily pain. "To each his sufferings," is the law of God, and we do not believe we children of the nineteenth century bear a bit lighter burden than our Anglo-Saxon ancestors. The lessons taught by anguish may be very valuable, the punishment of pain may be very salutary to many natures, but to seek more grief than comes by the law of nature is to return to the barbarism of the middle ages. Mr. White need not fear that pain is growing less, or will ever be banished from this earth. But if we go on growing daily more highly strung and more nervous in our organisation, we shall need anæsthetics to keep us from seeking death. Mental pain is a thousand times more bitter than bodily pain, and if we are decreasing the last, we are most certainly increasing the first.

